# Health-related quality of life and patterns of psychological vulnerability in autoimmune Addison’s disease: a systematic review

**DOI:** 10.3389/fendo.2026.1901783

**Published:** 2026-09-24

**Authors:** Orlando Silvestro, Ludovico Costa, Francesco Ferraù, Emanuele Maria Merlo, Salvatore Cannavò, Ulla Feldt-Rasmussen, Gabriella Martino

**Affiliations:** 1 Department of Clinical and Experimental Medicine, University of Messina, Messina, Italy; 2 Department of Human Pathology of Adulthood and Childhood “G. Barresi”, University of Messina, Messina, Italy; 3 Endocrinology Unit, University Hospital “G. Martino”, Messina, Italy; 4 Accademia Peloritana dei Pericolanti, Messina, Italy; 5 Department of Biomedical and Dental Sciences and Morphofunctional Imaging, University of Messina, Messina, Italy; 6 Department of Clinical Medicine, Faculty of Health and Medical Sciences, University of Copenhagen, Copenhagen, Denmark; 7 Department of Growth and Reproduction, Rigshospitalet, Copenhagen University Hospital, Copenhagen, Denmark

**Keywords:** autoimmune Addison’s disease, clinical psychology, executive functioning, fatigue, health-related quality of life, patient-reported outcomes, primary adrenal insufficiency, psychological distress

## Abstract

**Background:**

Autoimmune primary adrenal insufficiency (PAI) is the most common cause of Addison’s disease (AD) in Western countries and requires lifelong glucocorticoid and mineralocorticoid replacement. Despite biochemical control, many patients report persistent fatigue, functional limitations, and reduced well-being. However, evidence on health-related quality of life (HR-QoL) and psychopathological outcomes in autoimmune PAI remains fragmented and is often derived from mixed AD cohorts, limiting autoimmune-specific interpretation. This systematic review aimed to synthesise quantitative evidence on HR-QoL and psychological outcomes in adults with autoimmune PAI.

**Methods:**

Following PRISMA 2020 guidelines, a systematic search was conducted in PubMed, Scopus, and Web of Science from inception to February 2026 for English-language quantitative studies assessing HR-QoL and/or psychological outcomes in adults with autoimmune PAI or cohorts in which autoimmune aetiology of PAI was predominant. Study selection and data extraction were performed independently by two reviewers. Methodological quality was assessed using the NIH Study Quality Assessment Tools. The protocol was registered in PROSPERO (CRD420261322821).

**Results:**

Twenty-three studies met the inclusion criteria, including 1,764 participants with autoimmune PAI. Most investigations were observational, including eight cross-sectional and five case-control studies, while nine were interventional trials and one was a prospective observational study. HR-QoL was consistently reduced across studies, with the most prominent impairments observed in vitality, energy, and physical functioning domains. Female sex and greater autoimmune comorbidity were repeatedly associated with poorer perceived outcomes. Treatment-related findings were heterogeneous, with more consistent improvements observed in disease-specific HR-QoL following modified-release hydrocortisone or infusion strategies, while generic measures often showed limited change. Evidence on psychopathological symptoms was comparatively limited and mixed. Anxiety and depressive symptoms were generally mild and inconsistently elevated, while convergent findings emerged for mental fatigue, subjective executive functioning difficulties, and sexual distress in selected samples.

**Conclusions:**

Autoimmune PAI is associated with a persistent functional and psychological burden that is only partly captured by biochemical control. Integrating patient-reported outcomes into routine care may help identify individuals at risk of persistent impairment and support more comprehensive, multidisciplinary, patient-centred management strategies. Future longitudinal studies using harmonised outcome measures are needed to clarify trajectories, determinants, and potential targets for supportive interventions.

**Systematic review registration:**

https://www.crd.york.ac.uk/PROSPERO/, identifier CRD420261322821.

## 1 Introduction

Addison’s disease (AD) is a rare chronic endocrine disorder characterised by adrenal insufficiency of a variety of aetiologies. Autoimmune primary adrenal insufficiency (PAI) is a form of primary AD, resulting from autoimmune-mediated destruction of the adrenal cortex with progressive adrenal damage in most end-stage cases, leading to deficient secretion of all adrenal corticoid hormones (glucocorticoids, mineralocorticoids, and adrenal androgen production (1–3). In high-income countries, autoimmune PAI represents the dominant cause of AD, typically associated with circulating autoantibodies directed against steroidogenic enzymes. Among these, 21-hydroxylase autoantibodies (21OH-Ab) constitute a sensitive and specific marker of adrenal autoimmunity and may be detectable during preclinical disease stages (4, 5). Clinically, PAI follows an insidious course, with early non-specific manifestations, including weakness, weight loss, gastrointestinal symptoms, orthostatic hypotension, and salt craving (6–8). Therefore, diagnosis is often delayed, and a considerable proportion of patients are first identified during an acute adrenal crisis, a potentially life-threatening endocrine emergency characterised by severe hypotension, electrolyte imbalance, hypoglycaemia, and circulatory shock (9–11).

Epidemiologically, PAI is a rare disorder, with an estimated annual incidence of approximately 0.5-0.8 cases per 100.000 individuals and a prevalence ranging from 4 to 15 per 100.000, depending on population characteristics and ascertainment methods (3, 12, 13). The disease most commonly manifests in adulthood and shows a female predominance (14, 15). PAI frequently occurs in association with other autoimmune conditions or within autoimmune polyendocrine syndromes (APS), further increasing clinical complexity and long-term burden (4). The most frequent comorbid autoimmune disorders include autoimmune thyroid disease, type 1 diabetes mellitus, coeliac disease, and primary gonadal failure, with APS type 2 representing the most prevalent clinical configuration in adults (16–19).

Although survival has markedly improved following the introduction of hormone replacement therapy, PAI remains associated with substantial long-term morbidity, including recurrent hospitalisations, vulnerability to intercurrent illness, risk of adrenal crisis, and excess mortality (20–24). Importantly, accumulating evidence indicates that many patients continue to experience persistent symptoms and functional limitations despite apparently adequate endocrine replacement (14, 25–28). Residual manifestations such as fatigue, sleep disturbances, cognitive complaints, and limitations in daily activities are frequently reflected in reduced perceived health-related quality of life (HR-QoL) across physical, emotional, and social domains (29–31).

The persistence of impaired well-being in treated PAI has prompted increasing investigation into mechanisms linking endocrine dysregulation with psychological functioning. Proposed contributors include the non-physiological pattern of glucocorticoid replacement and its impact on circadian rhythmicity and stress-regulatory systems, as well as chronic low-grade inflammatory activity and autoimmune comorbidity, particularly in APS, which may influence energy regulation, stress responsivity, and emotional functioning (8, 32–34). Within this broader framework, psychological distress, particularly symptoms of anxiety and depression, has emerged as a clinically relevant dimension in PAI. Available data suggest that such symptoms may co-occur with somatic complaints and diminished HR-QoL, potentially affecting illness adjustment, daily functioning, and health-related behaviours (35–37).

Within this perspective, PAI may serve as a paradigm for a condition in which biological insufficiency, treatment-related factors, and psychological processes interact dynamically to shape patient-reported outcomes, including perceived quality of life and psychological well-being.

Although HR-QoL and psychological outcomes have been examined in previous reviews on AD, these syntheses have generally adopted a mixed-aetiology perspective, thereby limiting disease-specific interpretation and hindering the identification of mechanisms specifically related to PAI (38–41). To date, no systematic review has specifically synthesised quantitative evidence on HR-QoL and psychological outcomes with a dedicated focus on autoimmune PAI. The present systematic review, therefore, aims to systematically summarise and critically appraise available quantitative evidence on HR-QoL and psychological symptoms in adults with autoimmune PAI, to delineate the magnitude, consistency, and clinical relevance of these outcomes, as well as their potential interplay within the broader clinical phenotype of PAI. Clarifying these dimensions may contribute to a more comprehensive understanding of patient burden, support more nuanced clinical evaluation and monitoring, guide patient-centred management strategies, and identify priorities for future longitudinal research.

## 2 Methods

This systematic review was conducted in accordance with the Preferred Reporting Items for Systematic Reviews and Meta-Analyses (PRISMA) 2020 statement (42–44). The PRISMA 2020 checklist is provided in the Supplementary Materials (**Supplementary Tables 6**, **S7**). The review protocol was prospectively registered in the International Prospective Register of Systematic Reviews (PROSPERO; registration number: CRD420261322821).

### 2.1 Search strategy

A systematic literature search was performed in PubMed, Scopus, and Web of Science to identify studies evaluating HR-QoL and psychological outcomes in adults with autoimmune PAI. The search strategy combined controlled vocabulary terms (e.g., Medical Subject Headings in PubMed) and free-text keywords related to AD and PAI with terms referring to HR-QoL, depression, anxiety, and psychological distress. Equivalent search strategies were adapted for Scopus and Web of Science using database-specific syntax. No restrictions were applied to the publication year. The final search was conducted in February 2026. The complete search strings for each database are reported in the Supplementary Materials (**Supplementary Table 1**). No restrictions were applied to the geographical setting, and the reference lists of relevant reviews were also screened to ensure comprehensive coverage of the literature.

### 2.2 Inclusion and exclusion criteria

Studies were considered eligible if they met the following criteria: publication as full-text articles in peer-reviewed journals; availability in the English language; adoption of an empirical quantitative design; and assessment of perceived HR-QoL and/or mental health outcomes in adult samples (≥18 years), including patients with a confirmed diagnosis of autoimmune PAI. Given the rarity of autoimmune PAI, studies enrolling mixed AD populations were included only if at least one of the following conditions was satisfied: (a) results specific to autoimmune PAI were included separately (e.g., subgroups with autoimmune adrenalitis or 21OH-Ab positivity); or (b) autoimmune PAI was explicitly reported as the predominant aetiology in the sample (≥90%), even when results were not stratified by aetiology.

Studies were excluded if autoimmune PAI could not be clearly identified or represented a minority of the study population. Additional exclusion criteria comprised books, conference abstracts, narrative or systematic reviews, qualitative studies, and case reports.

### 2.3 Selection process and data collection

All records identified through the database searches were directly uploaded into Rayyan (45), where duplicate records were identified and removed. Titles and abstracts were independently screened in duplicate by two reviewers according to the predefined inclusion and exclusion criteria. Full-text articles deemed potentially eligible were subsequently assessed independently by the same reviewers. Any discrepancies at either stage were resolved through discussion and, when necessary, by consultation with a third reviewer until consensus was reached. In addition, backwards and forward snowballing were performed by examining the reference lists of all included studies and by identifying articles citing these studies through Scopus, to capture potentially relevant publications not retrieved through the database search. The study selection process was documented using a PRISMA 2020 flow diagram (**Figure 1**). Data extraction was performed independently by two reviewers using a standardised data extraction form. Data extracted from the included studies included authorship, year of publication, country, study design, sample characteristics, outcome measures assessing HR-QoL and/or mental health outcomes, and main findings (**Table 1**). When reported by eligible studies, additional patient-reported or functional domains closely related to HR-QoL or psychological outcomes, such as fatigue, sleep-related outcomes, executive functioning and sexual dysfunction, were also extracted and considered in the synthesis.

**Figure 1:**
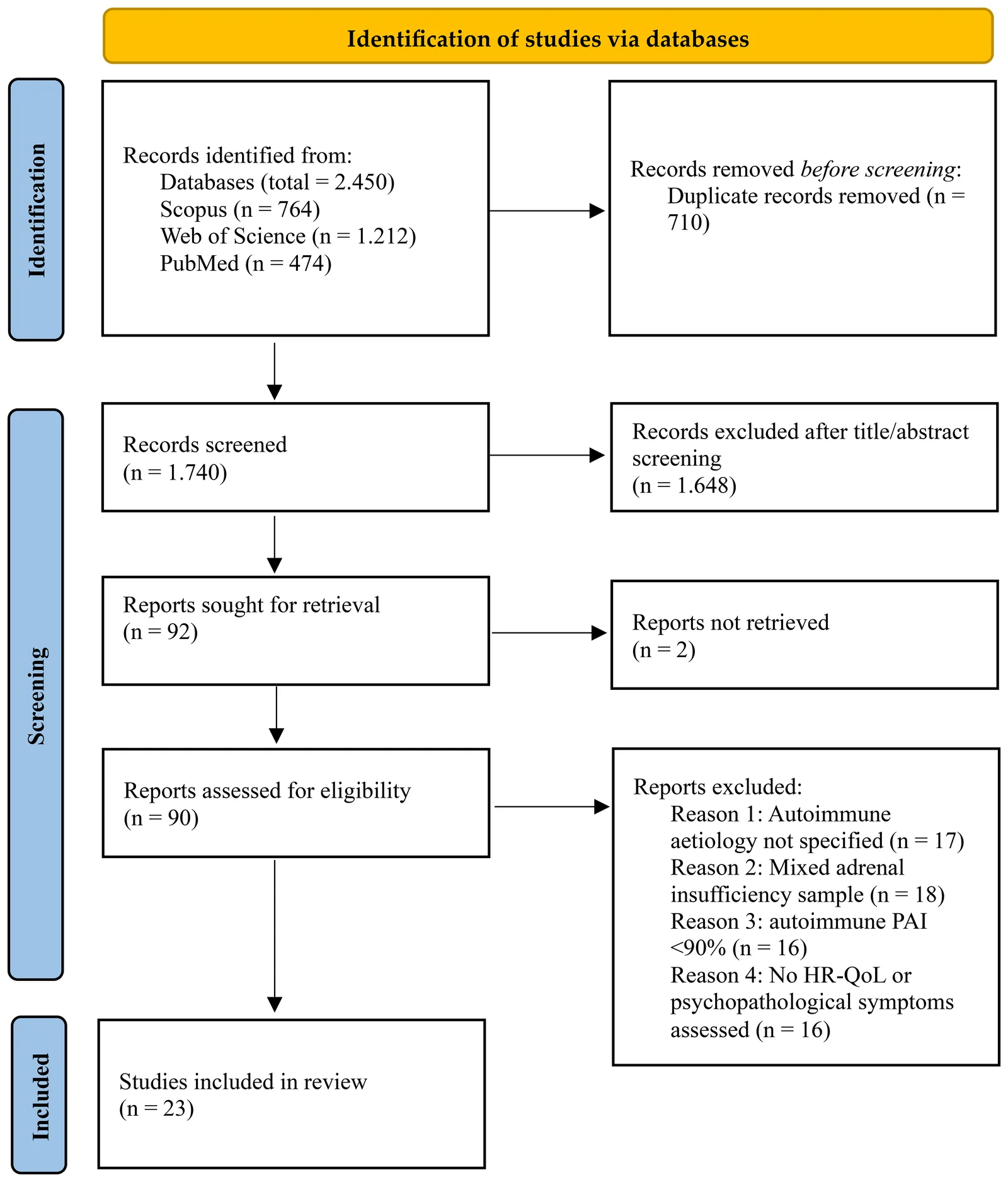
PRISMA flow diagram.

**Table 1:** Main characteristics of included studies.

Authors/year	Country	Study design	Sample	Measures	Main findings
46	Spain	Prospective interventional	12 PAI (11 autoimmune) 19 CG	NHP	Energy-related HR-QoL was reduced under both hydrocortisone regimens compared with reference data (p, 0.03; p, 0.001). Overall HR-QoL emerged only during the 10-5–5 mg/day regimen (p, 0.008), while adrenal insufficiency symptom scores did not differ between regimens.
47	Italy	Prospective interventional	20 PAI (ACA positivity)	AddiQoL-30	Disease-specific HR-QoL improved after switching from immediate-release to dual-release hydrocortisone (Group A: p, 0.005; Group B: p, 0.004). Improvements in ACTH levels and metabolic parameters were also reported.
48	Norway	Cross-sectional	494 PAI (421 21OH-Ab positivity)	RAND-36 AddiQoL-30 AddiQoL-8	Among 21OH-Ab-positive participants, men reported higher physical and mental HR-QoL than women (p < 0.001; p < 0.009). PCS was higher without autoimmune comorbidity and in younger age groups, whereas MCS was lowest in younger adults.
49	Sweden	Randomized crossover	15 PAI (21OH-Ab positivity) 7 CG	SF-36 FIS ESS	Hydrocortisone dose frequency did not clearly affect chemokine levels. Higher CXCL11 was associated with poorer physical HR-QoL (GH: p, 0.006; PCS: p, 0.004) and increased daytime sleepiness (ESS: p, 0.033).
50	Sweden	Randomized crossover	15 PAI	SF-36 FIS ESS	The four-dose regimen did not significantly improve HR-QoL compared with the two-dose regimen (MCS: p, 0.06; PCS: p, 0.17), nor fatigue or daytime sleepiness. The four-dose schedule was more frequently preferred (p, 0.03).
51	Norway	Cross-sectional	426 PAI (96% autoimmune aetiology)	SF-36	HR-QoL was lower than normative data, especially in vitality (50 [35-70] vs 60 [50-75]), and working disability was higher than in the general population (30% vs 11%). Type 1 diabetes, but not thyroid disease, was associated with poorer HR-QoL.
52	Sweden	Cross-sectional case-control	64 PAI 128 CG	SF-36 KSQ MFI-20	HR-QoL was poorer than in controls (p < 0.001), with greater fatigue (p, 0.007) and more sleep-related difficulties. Fatigue and non-restorative sleep were associated with lower HR-QoL, particularly in physical domains (p, 0.014).
53	Australia	Randomized crossover	10 PAI	SF-36 GHQ-28 FS GSRS AddiQoL-30	Baseline HR-QoL was mildly impaired, and 20% exceeded the clinical cut-off for psychological distress. No significant treatment-related differences emerged for fatigue, psychological distress, gastrointestinal symptoms, AddiQoL, or SF-36 outcomes.
54	Italy	Prospective interventional	19 PAI	AddiQoL-30	Disease-specific HR-QoL improved after 12 months of dual-release hydrocortisone, with AddiQoL increasing from 76.0 to 80.0 (p, 0.04). Improvements in metabolic parameters were also reported.
55	Italy	Prospective interventional	12 PAI	IIEF-15 BDI STAI	Sexual function was impaired at baseline, alongside elevated depression and anxiety scores. Sexual function and psychological symptoms improved after 2 months of glucocorticoid and mineralocorticoid replacement (p, 0.002).
56	Germany	Cross-sectional	132 PAI (29 autoimmune) 78 SAI	SF-36 GBB-24 HADS	The autoimmune PAI subgroup showed impaired HR-QoL in general health, vitality, social functioning, and role-emotional domains compared with normative data (p ≤ 0.05). Anxiety was higher than in controls (p, 0.032), whereas depression did not differ.
57	Finland	Cross-sectional	107 PAI	15D SF-36	HR-QoL was lower than in the general population (15D: p < 0.001). Female sex, lower education, longer disease duration, and autoimmune comorbidity were associated with poorer HR-QoL, whereas replacement regimen and healthcare follow-up level were not.
58	Norway	Cross-sectional	79 PAI (33 autoimmune; 9 APS-1; 37 APS-2)	SF-36 FQ	General health, vitality, and fatigue were worse than in normative data (all p ≤ 0.001). Physical role functioning was reduced mainly in women (p, 0.001), and working disability was higher than in the general population.
59	Germany	Cross-sectional	200 PAI	AddiQoL-36	Lower HR-QoL was associated with longer diagnostic delay, female sex, older age at disease onset and greater autoimmune comorbidity (all p ≤ 0.01). Hydrocortisone daily dose was not related to HR-QoL (p, 0.41).
60	Norway Sweden	Randomized crossover	33 PAI	AddiQoL-30 SF-36 PGWBI PSQI	Continuous subcutaneous hydrocortisone infusion improved disease-specific HR-QoL compared with conventional oral hydrocortisone (p, 0.012), mainly in vitality-related indices. Generic HR-QoL and subjective sleep quality showed less consistent improvement.
61	Norway Sweden Germany	Cross-sectional	192 PAI	AddiQoL-30 RAND-36	Residual glucocorticoid and mineralocorticoid production were present in 30.2% and 13.5% of cases, respectively, and were more frequent in men and in patients with shorter disease duration. Residual production was not associated with adrenal crisis frequency or HR-QoL (all p > 0.01).
62	Norway Sweden Germany	Cross-sectional case-control	43 PAI 43 CG	AddiQoL-30	Biomarker profiles differed between patients and controls, particularly among women. In exploratory analyses, vascular inflammation correlated with lower HR-QoL (p, 0.028) and more frequent adrenal crises (p, 0.006).
63	Norway	Randomized crossover	10 PAI	AddiQoL-30 SF-36	A single 10 mg pre-exercise hydrocortisone dose did not improve post-exercise HR-QoL compared with placebo, with no significant differences in AddiQoL-30 or SF-36 scores. No improvement was observed in short-term exercise performance.
64	Sweden	Cross-sectional case-control	52 PAI 70 CG	HADS MADRS BDEFS-SF	Depression and anxiety scores were slightly higher than in controls but remained within the non-clinical range. Total brain volume was smaller (p, 0.026), without clear associations with psychopathological symptom severity.
65	Sweden	Cross-sectional case-control	67 PAI 80 CG	HADS BDEFS-SF MFI	Cognitive performance was within the average range, and anxiety and depressive symptoms were not independently associated with executive functioning. Self-reported executive difficulties were associated with mental fatigue (p < 0.001) and lower glucocorticoid replacement dose.
66	Tunisia	Cross-sectional	58 PAI (90% autoimmune aetiology)	AddiQoL-30	Lower adherence to glucocorticoid replacement therapy was observed in 46% of patients. Adherence scores correlated with disease knowledge (p, 0.02) and AddiQoL total score (p, 0.01), whereas non-adherent patients had worse fatigue, emotional, and adrenal insufficiency symptom domain scores.
67	Italy	Cross-sectional case-control	22 PAI (20 APS-2) 23 CG	AddiQoL-30 FSFI-6 SDS	Sexual dysfunction and sexual distress were more frequent than in controls (p, 0.001; p, 0.007), with desire as the most affected domain (p < 0.001). Sexual dysfunction was associated with greater sexual distress and lower serum cortisol, but not with HR-QoL (p, 0.68).
68	Poland	Prospective observational	32 PAI	AddiQoL-30	Disease-specific HR-QoL was better in men than women (p, 0.011), whereas older age at diagnosis correlated with lower AddiQoL (r, -0.41, p, 0.038). Higher gonadotropin levels correlated with lower fatigue-related and emotional domain scores; replacement doses were not associated with AddiQoL.

15D, 15D instrument; 21OH-Ab, 21-Hydroxylase Autoantibodies; ACA, Adrenal Cortex Antibodies; ACTH, Adrenocorticotropic Hormone; AddiQoL, Addison’s Disease Quality of Life Questionnaire (30-, 8- and 36-item versions); APS, autoimmune polyendocrine syndrome (1 and 2); BDI, Beck Depression Inventory; BDEFS-SF, Barkley Deficits in Executive Functioning Scale - Short Form; CG, Control Group; CXCL11, C-X-C motif chemokine ligand 11; ESS, Epworth Sleepiness Scale; FIS, Fatigue Impact Scale; FQ, Fatigue Questionnaire; FS, Fatigue Scale; FSFI-6, Female Sexual Function Index - 6 item version; GH, General Health; GBB-24, Giessen Complaint List - 24 item short form; GHQ-28, General Health Questionnaire - 28 item version; GSRS, Gastrointestinal Symptom Rating Scale; HADS, Hospital Anxiety and Depression Scale; HR-QoL, Health-related Quality of Life; IIEF-15, International Index of Erectile Function-15; KSQ, Karolinska Sleep Questionnaire; MADRS, Montgomery–Åsberg Depression Rating Scale; MCS, Mental Component Summary; MFI-20, Multidimensional Fatigue Inventory (20 item version); NHP, Nottingham Health Profile; PAI, Primary Adrenal Insufficiency; PCS, Physical Component Summary; PGWBI, Psychological General Well-Being Index; PSQI, Pittsburgh Sleep Quality Index; RAND-36, RAND 36-Item Health Survey; SDS, Sexual Distress Scale; SF-36, 36-Item Short Form Health Survey; SAI, Secondary Adrenal Insufficiency; SSAI, Spielberger State Anxiety Inventory; STAI, Spielberger State-Trait Anxiety Inventory; Th1, T helper 1; Th2, T helper 2.

### 2.4 Quality assessment

The methodological quality of the included studies was independently assessed by two reviewers using the National Institutes of Health (NIH) Study Quality Assessment Tools, selecting the checklist appropriate to each study design. Any disagreements were resolved through discussion until a consensus was reached. Studies were classified as good, fair, or poor quality according to NIH guidelines. Detailed results of the quality appraisal are provided in the Supplementary Materials (**Supplementary Tables 2**-**S5**). The results of the quality assessment were considered in the interpretation of findings but were not used as exclusion criteria.

## 3 Results

The results of the literature search and study selection process are presented in **Figure 1**. The database search produced 2,450 records. After removal of duplicates, 1,740 records were screened, of which 1,648 were excluded as not relevant to the review objectives. A total of 92 articles were considered potentially eligible for full-text assessment; two reports could not be retrieved. The remaining 90 full-text articles were assessed for eligibility, of which 67 were excluded for the following reasons: unclear specification of autoimmune aetiology (n, 17), inclusion of mixed adrenal insufficiency samples without stratified data (n, 18), samples in which autoimmune PAI constituted less than 90% of the study population (n, 16), and absence of HR-QoL or psychopathological outcome measures (n, 16). In total, 23 studies met the inclusion criteria and were included in the qualitative synthesis. The main characteristics of the included studies and extracted outcomes are summarised in **Table 1**.

### 3.1 Characteristics of the included studies

The 23 studies included in the qualitative synthesis were published between 2002 and 2025 and conducted across multiple countries, predominantly in Europe. Studies were carried out in Norway (48, 51, 58, 60–63), Sweden (49, 50, 52, 64, 65), Germany (56, 59), Finland (57), Poland (68), Italy (47, 54, 55, 67), and Spain (46), with additional studies conducted in Australia (53) and Tunisia (66), reflecting a strong concentration of research activity in European populations.

Most investigations adopted observational designs. Eight studies employed cross-sectional methodologies, including normative comparisons and multicentre cohorts (48, 51, 56–59, 61, 66). Five studies used cross-sectional case-control designs comparing patients with autoimmune PAI to healthy controls (52, 62, 64, 65, 67). Nine studies adopted prospective interventional or randomised crossover designs. Most of these evaluated glucocorticoid replacement regimens or delivery modalities (46, 47, 49, 50, 53, 54, 60, 63), whereas Granata et al. (55) assessed combined glucocorticoid and mineralocorticoid replacement. Finally, one study employed a prospective observational design without experimental manipulation (68).

With respect to sample composition, most studies investigated autoimmune PAI defined populations (47, 49, 50, 52–55, 57, 59–65, 68). One early Norwegian cohort included exclusively autoimmune PAI but reported participants according to APS phenotypes (APS-1 and APS-2) without stratified analyses (58). In accordance with the predefined inclusion criteria, six additional studies enrolled AD cohorts: two provided specific autoimmune PAI subgroup analyses (48, 56), whereas four reported a predominantly autoimmune composition (≥90%) without aetiology-stratified analyses and were therefore retained (46, 51, 66, 67). Sample sizes varied considerably, ranging from small experimental samples (n, 10-15) in crossover trials (e.g., 49, 63) to registry-based cohorts exceeding 400 participants (51).

Regarding HR-QoL assessment, a wide range of instruments was used, reflecting variability in the conceptualisation and operationalisation of patient-reported outcomes across studies. Generic HR-QoL was most frequently measured using the 36-Item Short Form Health Survey (SF-36) or its equivalent RAND 36-Item Health Survey (RAND-36) (48–53, 56–58, 60, 61, 63). Additional generic measures included the 15D questionnaire (57), the Nottingham Health Profile (46), and the Psychological General Well-Being Index (60). Disease-specific HR-QoL was assessed using the Addison’s Disease Quality of Life Questionnaire (AddiQoL). Eleven studies employed the 30-item version (47, 48, 53, 54, 60–63, 66–68). The 36-item version was used in one study (59), and a short-form version in one study (48). Four studies administered both disease-specific and generic HR-QoL instruments concurrently (48, 53, 60, 63).

Mental health outcomes were evaluated in six studies using standardised self-report measures, indicating a comparatively limited representation of this domain. Anxiety and depressive symptoms were assessed using the Hospital Anxiety and Depression Scale (HADS) in three studies (56, 64, 65). The Beck Depression Inventory (BDI) and the Spielberger State-Trait Anxiety Inventory (STAI) were employed in one interventional study (55). Depressive symptom severity was further assessed using the Montgomery-Åsberg Depression Rating Scale (MADRS) in a recent case-control investigation (64), while general psychological distress was measured using the General Health Questionnaire-28 (GHQ-28) (53). HR-QoL and psychological distress were concurrently assessed in two studies (53, 56). One case-control study specifically addressed sexual distress alongside perceived HR-QoL (67). Finally, two recent case-control studies examined executive functioning complaints using the Barkley Deficits in Executive Functioning Scale - Short Form (BDEFS-SF), in conjunction with measures of affective symptoms (64, 65).

Overall, the included studies encompassed a total of 1.764 participants with an explicitly reported diagnosis of autoimmune PAI (e.g., 21OH-Ab positivity), as detailed in **Table 1**. One additional study included an AD sample in which 90% of cases were reported as autoimmune but did not provide the exact number of autoimmune participants and was therefore not included in the numerical total (66). Overall, considerable heterogeneity was observed across studies in terms of design, sample characteristics, outcome measures, and analytical approaches, which precluded direct quantitative synthesis. Regarding methodological quality, 22 studies were classified as fair and one controlled intervention study as good, while no study received poor rating (**Supplementary Tables 2**-**S5**). Overall, studies had clearly stated objectives, defined study populations and standardised outcome measures, whereas recurrent limitations included the absence of sample size justification and the lack of adjustment for confounding.

### 3.2 Health-related quality of life

A total of 20 studies assessed HR-QoL in patients with autoimmune PAI or samples predominantly including autoimmune PAI. Specifically, 11 adopted cross-sectional observational or case-control designs (48, 51, 52, 56–59, 61, 62, 66, 67), eight employed prospective interventional or randomized crossover methodologies (46, 47, 49, 50, 53, 54, 60, 63) and one study used a prospective observational design (68). Across the included evidence, six studies directly compared HR-QoL with population reference data or healthy control groups and consistently reported lower HR-QoL in patients with autoimmune PAI compared to healthy controls. The most consistently impaired domains were vitality, energy and general health perception, suggesting a central role of fatigue-related dimensions in the overall burden of autoimmune PAI (46, 51, 52, 56–58).

Twelve studies examined associations between HR-QoL and demographic, clinical, and disease-related characteristics within patient cohorts (48, 49, 51, 52, 57–59, 61, 62, 66–68). In this regard, sex-related differences in HR-QoL were repeatedly reported across seven studies, with female patients showing poorer outcomes, across both physical and mental health domains (48, 52, 57–59, 62, 68). Age and disease duration emerged as additional correlates of HR-QoL variability, with longer disease duration and older age at diagnosis associated with poorer perceived HR-QoL (57, 59, 68). In contrast, Didriksen et al. (48) reported that younger patients with autoimmune PAI exhibited poorer mental health compared to older patients.

Autoimmune comorbidity and overall disease burden were repeatedly linked to worse HR-QoL, particularly affecting physical functioning and vitality. Specifically, lower HR-QoL was observed in patients with coexisting autoimmune conditions such as type 1 diabetes (48, 51, 57, 59), whereas Erichsen et al. (51) found that thyroid disease alone did not consistently impact HR-QoL outcomes in a sample of predominantly autoimmune PAI.

Regarding fatigue and related features, four studies reported impairments in vitality or fatigue-related HR-QoL domains (46, 51, 58, 66). However, only one study tested its association with HR-QoL. This relative underrepresentation of fatigue as an explicitly analysed construct contrasts with its recurrent emergence across HR-QoL domains.

In a young adult autoimmune PAI cohort, Fletcher-Sandersjöö et al. (52) demonstrated that higher fatigue levels and poorer sleep quality were associated with lower HR-QoL, particularly in physical health domains. Additional clinical correlates were explored in a limited number of studies, suggesting a potential link between biological markers and patient-reported outcomes. Higher levels of inflammatory markers, including C-X-C motif chemokine ligand 11 (CXCL11), were associated with poorer physical HR-QoL and increased daytime sleepiness in a biomarker-focused crossover study (49), while exploratory analyses linked vascular inflammation markers to more frequent adrenal crises and lower disease-specific HR-QoL (62). However, these correlations did not remain statistically significant after correction for multiple testing and the study did not directly examine the association between adrenal crisis frequency and HR-QoL. One study addressed adherence-related factors, showing that age below 48 years and a history of adrenal crisis were independently associated with non-adherence to glucocorticoid replacement, while adherence scores were positively correlated with both disease knowledge and AddiQoL (66). In contrast, residual endogenous glucocorticoid or mineralocorticoid production was not associated with either HR-QoL or adrenal crisis frequency, despite being more frequent in male patients and those with shorter disease duration (61). Finally, one case-control study focusing on sexual dysfunction reported no significant association between HR-QoL and sexual dysfunction in a sample of women with APS-2 (67).

With regard to interventional evidence, eight prospective and randomised studies evaluated the impact of different hydrocortisone-based glucocorticoid replacement strategies on HR-QoL. Several studies did not detect statistically significant improvements in HR-QoL or fatigue-related outcomes between treatment conditions (49, 50, 53, 63), whereas Alonso et al. (46) reported a reduction in overall HR-QoL only under one hydrocortisone regimen (10-5–5 mg/day), with no differences observed in adrenal insufficiency symptom scores between regimens. In contrast, studies evaluating modified-release formulations or alternative delivery strategies more consistently reported improvements in disease-specific HR-QoL. Two prospective interventional studies showed that switching from conventional to dual-release hydrocortisone was associated with significant improvements in AddiQoL scores over follow-up (47, 54). Similarly, a randomised multicentre crossover study reported improvements in disease-specific HR-QoL, particularly in vitality-related indices, following continuous subcutaneous hydrocortisone infusion, although changes in generic HR-QoL measures were limited and sleep quality did not consistently improve (60). Three observational studies assessed the relationship between mineralocorticoid replacement and HR-QoL. Didriksen et al. (48) reported no significant differences in RAND-36 or AddiQoL scores according to mineralocorticoid dose or dosing regimen, while Kluger et al. (57) and Zdrojowy-Wełna et al. (68) observed no association between fludrocortisone dose and HR-QoL. Androgen replacement was examined in four further observational studies. Hahner et al. (56) reported no better subjective health status among patients receiving dehydroepiandrosterone (DHEA), and Kluger et al. (57) found no association between DHEA supplementation and HR-QoL among women. Similarly, Meyer et al. (59) observed lower AddiQoL scores in patients receiving DHEA, while Fletcher-Sandersjöö et al. (52) reported greater role limitations due to emotional problems among women receiving DHEA.

Taken together, the available evidence indicates that reduced HR-QoL represents a recurrent and clinically meaningful feature of autoimmune PAI. Despite heterogeneity in study design and assessment approaches, most investigations converge in documenting persistent impairments in perceived HR-QoL, particularly in vitality and physical domains, with considerable inter-individual variability and only partial responsiveness to current replacement strategies. These findings support the conceptualisation of HR-QoL as a key dimension of disease burden in autoimmune PAI, reflecting a complex interplay between biological insufficiency, treatment-related factors, and patient-reported experience, extending beyond biochemical indicators of disease control.

### 3.3 Psychopathological symptoms and psychological distress

A total of six studies examined psychopathological symptoms or psychological distress in patients with autoimmune PAI, highlighting comparatively limited evidence base in this domain. (53, 55, 56, 64, 65, 67). Four studies adopted a cross-sectional design, including three case-control investigations comparing patients with autoimmune PAI to healthy controls (64, 65, 67) and one study based on normative comparison (56). In addition, one prospective interventional study (55) and one randomised double-blind crossover study (53) assessed psychological outcomes in the context of treatment evaluation.

In a cross-sectional normative comparison, Hahner et al. (56) reported significantly higher anxiety symptoms in the autoimmune PAI subgroup, whereas no significant differences were observed in depressive symptoms. In interventional settings, Gagliardi et al. (53) observed that a subset of patients exceeded the clinical cut-off for psychological distress at baseline, although no significant differences emerged between treatment conditions for distress or HR-QoL outcomes. Granata et al. (55) documented elevated depression and anxiety scores in a sample of men with autoimmune PAI before the initiation of replacement therapy, followed by significant improvements after short-term combined glucocorticoid and mineralocorticoid treatment. In the same study, psychological symptoms severity was significantly associated with sexual dysfunction outcomes.

In a case-control study focusing on executive functioning in daily life, Van't Westeinde et al. (65) reported overall average cognitive performance and found that anxiety and depressive symptoms were not independently associated with executive functioning in multivariable analyses. Conversely, self-reported executive function difficulties, including emotion regulation domains, were associated with higher levels of mental fatigue and with lower glucocorticoid replacement dose. Among patients receiving immediate-release hydrocortisone, a higher number of adrenal crises was also associated with greater emotion-regulation difficulties. The relationship between adrenal crisis frequency and anxiety or depressive symptoms was not examined. In a sensitivity analysis, no differences were observed in the selected self-reported executive-function outcomes between women with and without DHEA supplementation. In a subsequent case-control neuroimaging study, Van't Westeinde et al. (64) observed slightly higher self-reported anxiety and depressive symptoms in autoimmune PAI than in controls, with differences approaching but not reaching conventional statistical significance. In the same cohort, a reduction in total brain volume was observed in patients with autoimmune PAI compared with healthy controls, suggesting the presence of subtle neurobiological alterations accompanying subclinical psychopathological features. Finally, a cross-sectional study in women with predominantly APS-2 reported a markedly higher prevalence of female sexual dysfunction and sex-related personal distress compared with healthy controls; desire was the most impaired domain, and sexual distress was inversely correlated with sexual function (67). However, among the relevant potential confounders, only age was accounted for through matching; menopausal status was reported descriptively, while androgen status and hormonal contraceptive use were not assessed.

Taken together, these findings suggest that psychopathological involvement in autoimmune PAI appears to be domain-specific rather than reflecting a uniform affective disorder profile, indicating a pattern of selective vulnerability. While anxiety and depressive symptoms were generally mild and inconsistently elevated compared with controls or reference values, convergent evidence pointed to impairments in selected domains, including executive functioning complaints, mental fatigue, elevated psychological distress in specific subgroups, sexual dysfunction and related distress, and subtle neurological alterations. However, the limited number of available studies and the heterogeneity of assessment tools should be considered when interpreting these findings.

## 4 Discussion

In the context of rare chronic medical conditions, HR-QoL and psychopathological symptoms represent clinically relevant outcomes that extend beyond biological markers (69–75). Accordingly, the assessment of perceived HR-QoL and psychological distress provides complementary information to biochemical indicators, capturing the subjective experience of illness and informing the need for person-centred approaches to care (76–80).

Within endocrinology, the physical and psychological burden associated with adrenal insufficiency has been documented across different aetiologies. However, autoimmune PAI represents a condition that warrants specific attention from both a biological and a functional perspective. Despite its clinical severity, the lifelong dependence on hormone replacement therapy and the persistent risk of adrenal crisis, autoimmune PAI has received comparatively less focused investigation regarding psychological outcomes compared with other forms of adrenal insufficiency (35, 36, 81, 82). The present systematic review specifically addresses this gap by synthesising evidence referring to autoimmune aetiology.

The findings of this review indicate that HR-QoL appears to be consistently impaired in patients with autoimmune PAI. Convergent results emerge from studies comparing patients with normative population data as well as from investigations including healthy control groups. These findings are in line with previous evidence showing that adrenal insufficiency, even when adequately treated, may substantially compromise everyday functioning (83–85). Importantly, the impairment appears to be structured around specific domains, particularly vitality, energy, and physical role functioning, rather than reflecting a diffuse or unspecific reduction in well-being. This pattern suggests that functional limitations and persistent fatigue constitute core components of the subjective disease burden (86–88). Consistent with this functional interpretation, recent nationwide register-based studies indicate that autoimmune PAI is associated with increased work loss and substantial socioeconomic consequences compared with matched population comparators (89, 90).

With respect to treatment-related outcomes, the available evidence appears heterogeneous. Approximately half of the interventional studies did not report significant changes in perceived HR-QoL following different replacement regimens, whereas others documented meaningful improvements. Such variability is likely attributable to differences in treatment strategies, patient characteristics, duration of follow-up, and the use of generic versus disease-specific HR-QoL instruments. Notably, the most consistent benefits were observed with replacement strategies designed to more closely reproduce physiological cortisol exposure.

One possible explanation for the more favourable findings observed with dual-release hydrocortisone and continuous subcutaneous hydrocortisone infusion is their ability to better mimic the physiological circadian cortisol rhythm. Compared with conventional immediate-release regimens, these approaches may reduce periods of relative hypo- and hypercortisolism throughout the day, thereby limiting cortisol fluctuations that have been implicated in fatigue, impaired vitality, sleep disturbances, and reduced daytime functioning. In addition, simplified dosing schedules may lessen treatment burden and improve adherence, although these aspects have not been systematically evaluated. Overall, these observations support the concept that the quality of cortisol replacement, rather than glucocorticoid dose alone, may be an important determinant of patient-reported outcomes. However, this hypothesis requires confirmation in adequately powered prospective studies.

Beyond dual-release hydrocortisone, newer modified-release formulations such as Chronocort, which are specifically designed to reproduce the physiological overnight rise and early morning cortisol peak, have also shown promising improvements in fatigue, morning functioning, and patient-reported quality of life in recent studies. Although these data were not eligible for inclusion in the present systematic review, they further support the concept that more physiological cortisol replacement may translate into clinically meaningful improvements in everyday functioning and HR-QoL (91).

In contrast, evidence for other hormonal replacement strategies remains limited. Mineralocorticoid replacement has been evaluated only in observational analyses of dose or treatment regimen, which consistently found no association with HR-QoL (48, 57, 68). Although Granata et al. (55) reported improvements in anxiety and depression following combined glucocorticoid and mineralocorticoid replacement, the simultaneous introduction of both treatments precluded attribution of these effects specifically to fludrocortisone. Similarly, DHEA replacement has been investigated only in observational studies assessing HR-QoL and in a sensitivity analysis of self-reported executive functioning. Findings were inconsistent across HR-QoL domains, treated subgroups were generally small, and no included study specifically evaluated the effects of DHEA on anxiety, depression, or general psychological distress (52, 56, 57, 59, 65). Therefore, the currently available evidence does not allow the independent contribution of mineralocorticoid or androgen replacement to HR-QoL and psychological outcomes to be established.

Evidence concerning glucocorticoid overreplacement was similarly limited. None of the included studies operationalised overreplacement or examined it as an independent determinant of HR-QoL or adverse psychological outcomes in autoimmune PAI. Nevertheless, overtreatment remains clinically relevant, as current guidelines recommend monitoring for signs of glucocorticoid excess, including weight gain, insomnia, and peripheral oedema, while broader evidence from adrenal insufficiency suggests that hydrocortisone exposure may influence perceived health and depressive symptoms (1, 41). Prospective studies are needed both to clarify the independent effects of mineralocorticoid and androgen replacement and to assess the potential contribution of glucocorticoid overreplacement to HR-QoL and psychological functioning.

These results may also reflect the fact that patient-reported well-being in autoimmune PAI is shaped not only by hormonal substitution but also by the broader demands of chronic disease management, including persistent symptoms and functional constraints. In this sense, HR-QoL operates as an integrative patient-reported indicator, capturing aspects of daily experience that may remain clinically salient even when standard endocrine targets are achieved (92, 93).

Considering factors associated with HR-QoL in autoimmune PAI, the included studies consistently showed poorer perceived outcomes in female patients. This sex disparity has been reported across both generic and disease-specific instruments and appears to be primarily driven by the physical dimension of HR-QoL. Although causal mechanisms cannot be established from the available evidence, several explanations are plausible, including differences in comorbidity burden, endocrine milieu (e.g., relative adrenal androgen deficiency), and differential exposure to functional role demands that may amplify the impact of symptom fluctuations on daily life (94–96). Similar patterns were observed with respect to age and disease duration, with poorer HR-QoL generally reported among older patients and those with longer-standing disease. This gradient is consistent with cumulative burden models in chronic conditions, whereby ageing-related decline and accrued comorbidity progressively constrain physical functioning and perceived vitality (97). Notably, one large cohort study found that younger age was associated with poorer perceived mental health, with the lowest mental component scores observed in patients in early adulthood compared with older age groups (48). This age-specific pattern contrasts with the overall gradient observed for physical HR-QoL and disease duration and suggests that mental well-being in autoimmune PAI may be differentially influenced by developmental stage. In young adults, the demands of lifelong treatment and disease management may intersect with ongoing educational, occupational, and relational transitions, potentially amplifying their impact on perceived mental health. This hypothesis is consistent with broader evidence from chronic illness research indicating that earlier adulthood represents a particularly vulnerable period for HR-QoL outcomes (98, 99).

Collectively, these observations highlight the importance of longitudinal studies capable of capturing life-course dynamics in autoimmune PAI. Furthermore, several studies reported an association between autoimmune comorbidity and poorer HR-QoL, particularly in physical domains, although this pattern was not uniform across all conditions and samples (48, 51, 57, 59). Taken together, these findings suggest that perceived well-being in autoimmune PAI is influenced not only by adrenal insufficiency itself, but also by the broader multimorbid context in which the disease frequently occurs, including additional autoimmune diagnoses and their functional consequences. Although autoimmune thyroid disease is by far the most prevalent autoimmune condition (100) and is often associated with impaired HR-QoL (101), it is unlikely to substantially contribute to the reduced HR-QoL observed in patients with PAI (51). However, it may serve as an important marker of the broader autoimmune context.

Evidence regarding the specific contribution of APS-related comorbidities to psychopathological symptoms or psychological distress was less direct. The most informative comparison was provided by Løvås et al. (58), who reported that patients with APS-1 or APS-2 tended to have poorer subjective health status than those with isolated autoimmune PAI, with impairments in social functioning and role-emotional domains mainly observed in APS subgroups. However, mental fatigue was increased across disease categories, suggesting that this dimension may be more closely related to adrenal insufficiency itself than to APS-related comorbidity. In line with this interpretation, Hahner et al. (56) found that impaired subjective health status and increased anxiety persisted after excluding concomitant diseases, including among patients with isolated autoimmune PAI. Zamponi et al. (67) reported increased female sexual dysfunction and sex-related personal distress in a predominantly APS-2 female sample, but the absence of a comparison with isolated autoimmune PAI prevents attribution of these findings to APS-2 specifically. Overall, available evidence suggests that APS-related comorbidity may contribute to selected social, emotional, sexual, and functional aspects of patient-reported burden, whereas its independent association with psychopathological symptoms or psychological distress remains insufficiently established.

From a clinical perspective, this highlights the importance of interpreting HR-QoL data within the overall health profile of each patient, considering comorbidity load and long-term illness trajectories (102). This perspective should also extend beyond APS-related autoimmunity. In the present synthesis, autoimmune comorbidity was the most consistently documented dimension. However, poorer perceived health outcomes were also associated with markers of cumulative clinical and functional burden, including age, disease duration, fatigue, sleep quality, adherence-related factors and psychopathological symptoms. Evidence from adrenal insufficiency and chronic disease research indicate that perceived health and everyday functioning are shaped by the combined impact of coexisting medical conditions, persistent symptoms and psychopathological comorbidities, rather than by endocrine replacement or autoimmune status alone (82, 84, 87, 97, 103). Accordingly, HR-QoL and psychological well-being in autoimmune PAI should be interpreted within a comprehensive clinical framework encompassing medical comorbidity, psychopathological morbidity, persistent symptoms and functional burden.

Concerning psychopathological symptoms, the findings of this review suggest that psychological involvement in autoimmune PAI does not conform to a disorder-specific profile but rather manifests through heterogeneous and context-dependent vulnerabilities. Across studies, anxiety, depressive symptoms, and general psychological distress showed variable patterns, with elevations observed in specific samples, phases of disease, or functional domains, rather than as consistently pervasive patterns (53, 56). Notably, convergent signals emerged in areas such as mental fatigue, subjective executive functioning difficulties, emotional regulation complaints, and sexual distress, indicating that psychological burden in autoimmune PAI may be more closely linked to functional and experiential dimensions than to categorical psychopathology (65, 67). This interpretation supports a dimensional and patient-centred conceptualisation of psychological outcomes in autoimmune PAI, emphasising the clinical relevance of targeted, domain-specific assessment over assumptions of a global affective disorder. Future longitudinal and methodologically standardised studies are needed to clarify how these vulnerabilities evolve and interact with disease course and treatment.

Evidence specifically linking adrenal crisis frequency to psychological outcomes was limited. In the young adult cohort examined by Van't Westeinde et al. (65), a higher number of adrenal crises was associated with greater self-reported emotion-regulation difficulties, but only among patients receiving immediate-release hydrocortisone. Adrenal crisis frequency was not analysed as a determinant of anxiety or depressive symptoms. Additional findings were indirect. Sævik et al. (62) identified exploratory correlations between vascular inflammation markers, adrenal crisis frequency, and lower disease-specific HR-QoL, although these associations did not remain significant after correction for multiple testing. Yazidi et al. (66) found that relatively younger age and a history of adrenal crisis were associated with non-adherence, while adherence was correlated with disease-specific HR-QoL. Taken together, these cross-sectional findings do not establish whether recurrent adrenal crises independently contribute to anxiety, depression or reduced HR-QoL. Prospective studies are needed to distinguish their potential direct psychological impact from the influence of treatment adherence, replacement adequacy and broader disease burden.

Overall, these findings support the clinical utility of adopting a multidisciplinary and patient-centred model of care in autoimmune PAI. Alongside endocrine monitoring, routine assessment of patient-reported outcomes may help identify individuals at higher risk of persistent functional burden (103, 104). In practical terms, screening may be prioritised for recurrent fatigue and sleep problems, psychopathological symptoms, cognitive and executive complaints and sexual distress when clinically indicated. Early identification of these profiles could facilitate timely referral to psychological support and tailored interventions aimed at improving illness adjustment, self-management, and HR-QoL (105–107). Future research should prioritise longitudinal designs to clarify trajectories of HR-QoL and psychological symptoms over time, and to test prospective associations with clinical variables such as treatment regimens, comorbidity burden within APS and adrenal crisis frequency. Methodological harmonisation across studies, particularly regarding the choice of validated HR-QoL and mental health instruments and the reporting of specific autoimmune PAI data, would also strengthen comparability and enable quantitative synthesis in subsequent reviews.

This review has several limitations. First, the included studies were highly heterogeneous in design (cross-sectional cohorts, case-control studies, and interventional crossover trials), outcomes, and analytic approaches, which prevented meta-analysis and limited comparability across findings. In addition, the literature search was conducted in PubMed, Scopus, and Web of Science and did not include PsycINFO. Notwithstanding this non-inclusion, the broad biomedical and multidisciplinary coverage of the databases searched provided a solid basis for identifying evidence relevant to the interdisciplinary focus of this review.

More broadly, it is important to acknowledge the inherent challenges in generating high-quality evidence in rare conditions such as autoimmune PAI. In particular, the feasibility of adequately powered randomised controlled trials is often limited, and the available evidence frequently relies on small samples, observational designs, or heterogeneous interventional studies. These structural constraints represent an intrinsic limitation of the field and should be considered when interpreting the strength and generalisability of the findings.

Second, HR-QoL and psychological outcomes were assessed using a wide range of instruments; in particular, psychopathological symptoms were evaluated in only a small subset of studies, reducing confidence in the consistency of mental health estimates. Third, although only studies with specific autoimmune PAI data or a predominantly autoimmune composition (≥90%) were included, the lack of aetiology-stratified reporting in some studies limited the possibility of determining whether observed HR-QoL and psychological outcome patterns were fully attributable to autoimmune PAI alone. This approach was adopted to balance methodological rigour with feasibility, given the rarity of autoimmune PAI and the limited availability of studies exclusively focused on this condition. Finally, the restriction to English-language full-text articles may have contributed to publication and language bias.

Taken together, evidence synthesised in this review delineates autoimmune PAI as a chronic condition characterised by a persistent functional and psychological burden that is only partially captured by biomedical indicators. Notably, the persistence of reduced HR-QoL despite apparently adequate hormone replacement highlights a clinically relevant dissociation between biochemical control and subjective disease burden (108). Across heterogeneous study designs and assessment tools, reduced HR-QoL emerged as a stable and recurrent feature, while psychopathological involvement appeared more nuanced, domain-specific, and closely intertwined with everyday functioning.

This pattern underscores the importance of conceptualising patient experience in autoimmune PAI along a continuum of functional and psychological adaptation, in which fatigue, vitality, cognitive complaints and emotional regulation represent key dimensions of illness impact. Within this framework, HR-QoL and psychological outcomes should be regarded not as secondary endpoints, but as integral components of the overall disease burden in autoimmune PAI, reflecting aspects of patient experience that remain clinically relevant despite adequate endocrine replacement.

## 5 Conclusions

This systematic review indicates that autoimmune PAI is associated with a persistent patient-reported burden that is only partly captured by biomedical indices. Across study designs and instruments, reduced HR-QoL emerged as a consistent and clinically meaningful feature, with the most recurrent impairments involving vitality, energy, and physical functioning. By contrast, evidence on psychopathological symptoms remains comparatively limited and methodologically heterogeneous. Available findings do not support a uniform affective disorder profile, but rather suggest domain-specific vulnerabilities, including mental fatigue, subjective executive functioning difficulties, and psychological distress in selected samples. Incorporating patient-reported outcomes into routine endocrine follow-up may help identify patients with persistent functional and psychological impairment and guide timely multidisciplinary management. Future research should prioritise longitudinal designs, harmonised assessment strategies, and more consistent aetiology- and comorbidity-stratified reporting to clarify trajectories, determinants, and potential targets for supportive interventions.

## Data Availability

The raw data supporting the conclusions of this article will be made available by the authors, without undue reservation.
