# Supplementary material for: Health-related quality of life and patterns of psychological vulnerability in autoimmune Addison’s disease: a systematic review
Source: Front Endocrinol (Lausanne). 2026 Sep 24;17:1901783. doi: 10.3389/fendo.2026.1901783 (PMC13645576; doi:10.3389/fendo.2026.1901783)
Supplement: Supplementary file 1 [file Table1.docx]

**Supplementary Material**

**Table S1.** Electronic search strategies

| **Database** | **Search strategy** |
| --- | --- |
| PubMed | (“Addison Disease”[Mesh] OR addison*[tiab] OR addisonian[tiab] OR “Addison's disease”[tiab] OR “primary adrenal insufficiency”[tiab] OR “autoimmune adrenalitis”[tiab]) AND (“Quality of Life”[Mesh] OR “Mental Disorders”[Mesh] OR “Depressive Disorder”[Mesh] OR “Anxiety Disorders”[Mesh] OR “Stress, Psychological”[Mesh] OR “quality of life”[tiab] OR “health-related quality of life”[tiab] OR HRQoL[tiab] OR wellbeing[tiab] OR “well-being”[tiab] OR depress*[tiab] OR anxi*[tiab] OR distress[tiab] OR “psychological stress”[tiab]) |
| Scopus | (“Addison Disease” OR addison* OR addisonian OR “Addison's disease” OR “primary adrenal insufficiency” OR “autoimmune adrenalitis”) AND (“Quality of Life” OR “Mental Disorders” OR “Depressive Disorder” OR “Anxiety Disorders” OR “Stress, Psychological” OR “quality of life” OR “health-related quality of life” OR HRQoL OR wellbeing OR “well-being” OR depress* OR anxi* OR distress OR “psychological stress”) |
| Web of Science | (“Addison Disease” OR addison* OR addisonian OR “Addison's disease” OR “primary adrenal insufficiency” OR “autoimmune adrenalitis”) AND (“Quality of Life” OR “Mental Disorders” OR “Depressive Disorder” OR “Anxiety Disorders” OR “Stress, Psychological” OR “quality of life” OR “health-related quality of life” OR HRQoL OR wellbeing OR “well-being” OR depress* OR anxi* OR distress OR “psychological stress”) |

**Table S2.** Quality appraisal for Observational Cohort and Cross-sectional Studies

| **Study** | **Year** | **1. Was the study question or objective clearly stated?** | **2. Was the study population clearly specified and defined?** | **3. Was the participation rate of eligible persons at least 50%?** | **4. Were all the subjects selected or recruited from the same or similar populations (including the same time period)? Were inclusion and exclusion criteria for being in the study prespecified and applied uniformly to all participants?** | **5. Was a sample size justification, power description, or variance and effect estimates provided?** | **6. For the analyses in this paper, were the exposure(s) of interest measured prior to the outcome(s) being measured?** | **7. Was the timeframe sufficient so that one could reasonably expect to see an association between exposure and outcome if it existed?** | **8. For exposures that can vary in amount or level, did the study examine different levels of the exposure as related to the outcome (e.g., categories of exposure, or exposure measured as continuous variable)?** | **9. Were the exposure measures (independent variables) clearly defined, valid, reliable, and implemented consistently across all study participants?** | **10. Was the exposure(s) assessed more than once over time?** | **11. Were the outcome measures (dependent variables) clearly defined, valid, reliable, and implemented consistently across all study participants?** | **12. Were the outcome assessors blinded to the exposure status of participants?** | **13. Was loss to follow-up after baseline 20% or less?** | **14. Were key potential confounding variables measured and adjusted statistically for their impact on the relationship between exposure(s) and outcome(s)?** | **Quality Raging (Good, Fair or Poor** |
| --- | --- | --- | --- | --- | --- | --- | --- | --- | --- | --- | --- | --- | --- | --- | --- | --- |
| Didriksen et al. | 2021 | Y | Y | CD | Y | N | N | N | Y | Y | N | Y | NA | NA | CD | Fair |
| Erichsen et al. | 2009 | Y | Y | Y | Y | N | N | N | Y | Y | N | Y | NA | NA | CD | Fair |
| Hahner et al. | 2007 | Y | Y | CD | Y | N | N | N | Y | Y | N | Y | NA | NA | Y | Fair |
| Kluger et al. | 2014 | Y | Y | CD | Y | N | N | N | Y | Y | N | Y | NA | NA | Y | Fair |
| Løvås et al. | 2002 | Y | Y | Y | Y | N | N | N | Y | Y | N | Y | NA | NA | CD | Fair |
| Meyer et al. | 2013 | Y | Y | CD | Y | N | N | N | Y | Y | N | Y | NA | NA | Y | Fair |
| Sævik et al. | 2020 | Y | Y | CD | Y | N | Y | Y | Y | Y | NA | Y | NA | NA | Y | Fair |
| Yazidi et al. | 2023 | Y | Y | CD | Y | N | N | N | Y | Y | N | Y | NA | NA | Y | Fair |
| Zdrojowy-Wełna et al. | 2023 | Y | Y | Y | Y | N | N | N | Y | Y | N | Y | NA | NA | N | Fair |

**Note.** CD, cannot determine; Y, yes; NA, not applicable; N, no; NR, not reported. A study will be rated as “Good” if it receives a “Y" response for ≥80% of the applicable NIH critical appraisal questions, “Fair” for 50%-79%, and “Poor” for ≤50%.

**Table S3.** Quality appraisal of Case-Control Studies

| **Study** | **Year** | **1. Was the research question or objective in this paper clearly stated and appropriate?** | **2. Was the study population clearly specified and defined?** | **3. Did the authors include a sample size justification?** | **4. Were controls selected or recruited from the same or similar population that gave rise to the cases (including the same timeframe)?** | **5. Were the definitions, inclusion and exclusion criteria, algorithms or processes used to identify or select cases and controls valid, reliable, and implemented consistently across all study participants?** | **6. Were the cases clearly defined and differentiated from controls?** | **7. If less than 100 percent of eligible cases and/or controls were selected for the study, were the cases and/or controls randomly selected from those eligible?** | **8. Was there use of concurrent controls?** | **9. Were the investigators able to confirm that the exposure/risk occurred prior to the development of the condition or event that defined a participant as a case?** | **10. Were the measures of exposure/risk clearly defined, valid, reliable, and implemented consistently (including the same time period) across all study participants?** | **11. Were the assessors of exposure/risk blinded to the case or control status of participants?** | **12. Were key potential confounding variables measured and adjusted statistically in the analyses? If matching was used, did the investigators account for matching during study analysis?** | **Quality Raging (Good, Fair or Poor** |
| --- | --- | --- | --- | --- | --- | --- | --- | --- | --- | --- | --- | --- | --- | --- |
| Fletcher-Sandersjöö et al. | 2025 | Y | Y | N | Y | Y | Y | CD | Y | NA | Y | NA | Y | Fair |
| Sævik et al. | 2023 | Y | Y | N | Y | Y | Y | CD | Y | CD | Y | CD | Y | Fair |
| Van’t Westeinde et al. | 2023 | Y | Y | Y | Y | Y | Y | CD | Y | CD | Y | CD | Y | Fair |
| Van’t Westeinde et al. | 2022 | Y | Y | N | Y | Y | Y | CD | Y | CD | Y | CD | N | Fair |
| Zamponi et al. | 2021 | Y | Y | N | Y | Y | Y | CD | Y | NA | Y | CD | N | Fair |

**Note.** CD, cannot determine; Y, yes; NA, not applicable; N, no; NR, not reported. A study will be rated as “Good” if it receives a “Y" response for ≥80% of the applicable NIH critical appraisal questions, “Fair” for 50%-79%, and “Poor” for ≤50%.

**Table S4.** Quality appraisal of Before-After (Pre-Post) Studies With No Control Group

| **Study** | **Year** | **1. Was the study question or objective clearly stated?** | **2. Were eligibility/ selection criteria for the study population prespecified and clearly described?** | **3. Were the participants in the study representative of those who would be eligible for the test/service/ intervention in the general or clinical population of interest?** | **4.  Were all eligible participants that met the prespecified entry criteria enrolled?** | **5. Was the sample size sufficiently large to provide confidence in the findings?** | **6. Was the test/service/ intervention clearly described and delivered consistently across the study population?** | **7. Were the outcome measures prespecified, clearly defined, valid, reliable, and assessed consistently across all study participants?** | **8. Were the people assessing the outcomes blinded to the participants' exposures/ interventions?** | **9. Was the loss to follow-up after baseline 20% or less? Were those lost to follow-up accounted for in the analysis?** | **10. Did the statistical methods examine changes in outcome measures from before to after the intervention? Were statistical tests done that provided p values for the pre-to-post changes?** | **11. Were outcome measures of interest taken multiple times before the intervention and multiple times after the intervention (i.e., did they use an interrupted time-series design)?** | **12. If the intervention was conducted at a group level (e.g., a whole hospital, a community, etc.) did the statistical analysis take into account the use of individual-level data to determine effects at the group level?** | **Quality Raging (Good, Fair or Poor** |
| --- | --- | --- | --- | --- | --- | --- | --- | --- | --- | --- | --- | --- | --- | --- |
| Alonso et al. | 2004 | Y | Y | N | CD | N | Y | Y | NR | Y | Y | N | NA | Fair |
| Delle Cese et al. | 2021 | Y | Y | N | CD | N | Y | Y | NR | Y | Y | N | NA | Fair |
| Giordano et al. | 2016 | Y | Y | Y | Y | N | Y | Y | N | Y | Y | N | NA | Fair |
| Granata et al. | 2013 | Y | Y | N | CD | N | Y | Y | NR | Y | Y | N | NA | Fair |

**Note.** CD, cannot determine; Y, yes; NA, not applicable; N, no; NR, not reported. A study will be rated as “Good” if it receives a “Y" response for ≥80% of the applicable NIH critical appraisal questions, “Fair” for 50%-79%, and “Poor” for ≤50%.

**Table S5.** Quality appraisal of Controlled Intervention Studies

| **Study** | **Year** | **1. Was the study described as randomized, a randomized trial, a randomized clinical trial, or an RCT?** | **2. Was the method of randomization adequate (i.e., use of randomly generated assignment)?** | **3. Was the treatment allocation concealed (so that assignments could not be predicted)?** | **4. Were study participants and providers blinded to treatment group assignment?** | **5. Were the people assessing the outcomes blinded to the participants' group assignments?** | **6. Were the groups similar at baseline on important characteristics that could affect outcomes (e.g., demographics, risk factors, co-morbid conditions)?** | **7. Was the overall drop-out rate from the study at endpoint 20% or lower of the number allocated to treatment?** | **8. Was the differential drop-out rate (between treatment groups) at endpoint 15 percentage points or lower?** | **9. Was there high adherence to the intervention protocols for each treatment group?** | **10. Were other interventions avoided or similar in the groups (e.g., similar background treatments)?** | **11. Were outcomes assessed using valid and reliable measures, implemented consistently across all study participants?** | **12. Did the authors report that the sample size was sufficiently large to be able to detect a difference in the main outcome between groups with at least 80% power?** | **13. Were outcomes reported or subgroups analyzed prespecified (i.e., identified before analyses were conducted)?** | **14. Were all randomized participants analyzed in the group to which they were originally assigned, i.e., did they use an intention-to-treat analysis?** | **Quality Raging (Good, Fair or Poor** |
| --- | --- | --- | --- | --- | --- | --- | --- | --- | --- | --- | --- | --- | --- | --- | --- | --- |
| Ekman et al. | 2014 | Y | NR | NR | Y | Y | Y | Y | NA | Y | Y | Y | CD | Y | CD | Fair |
| Ekman et al. | 2012 | Y | NR | NR | Y | Y | Y | Y | NA | Y | Y | Y | CD | Y | CD | Fair |
| Gagliardi et al. | 2014 | Y | NR | Y | Y | Y | Y | N | NA | Y | Y | Y | N | Y | N | Fair |
| Øksnes et al. | 2014 | Y | Y | CD | N | CD | Y | Y | NA | Y | Y | Y | Y | Y | CD | Fair |
| Simunkova et al. | 2016 | Y | Y | Y | Y | Y | Y | Y | NA | Y | Y | Y | NR | Y | NA | Good |

**Note.** CD, cannot determine; Y, yes; NA, not applicable; N, no; NR, not reported. A study will be rated as “Good” if it receives a “Y" response for ≥80% of the applicable NIH critical appraisal questions, “Fair” for 50%-79%, and “Poor” for ≤50%.

**Table S6.** PRSIMA Abstract checklist

| **Section and Topic** | **Item #** | **Checklist item** | **Reported (Yes/No)** |
| --- | --- | --- | --- |
| **TITLE** | | |  |
| Title | 1 | Identify the report as a systematic review. | Provided |
| **BACKGROUND** | | |  |
| Objectives | 2 | Provide an explicit statement of the main objective(s) or question(s) the review addresses. | Background section |
| **METHODS** | | |  |
| Eligibility criteria | 3 | Specify the inclusion and exclusion criteria for the review. | Methods section |
| Information sources | 4 | Specify the information sources (e.g. databases, registers) used to identify studies and the date when each was last searched. | Methods section |
| Risk of bias | 5 | Specify the methods used to assess risk of bias in the included studies. | Methods section |
| Synthesis of results | 6 | Specify the methods used to present and synthesise results. | Methods section |
| **RESULTS** | | |  |
| Included studies | 7 | Give the total number of included studies and participants and summarise relevant characteristics of studies. | Results section |
| Synthesis of results | 8 | Present results for main outcomes, preferably indicating the number of included studies and participants for each. If meta-analysis was done, report the summary estimate and confidence/credible interval. If comparing groups, indicate the direction of the effect (i.e. which group is favoured). | Results section |
| **DISCUSSION** | | |  |
| Limitations of evidence | 9 | Provide a brief summary of the limitations of the evidence included in the review (e.g. study risk of bias, inconsistency and imprecision). | Conclusion section |
| Interpretation | 10 | Provide a general interpretation of the results and important implications. | Conclusion section |
| **OTHER** | | |  |
| Funding | 11 | Specify the primary source of funding for the review. | Not applicable |
| Registration | 12 | Provide the register name and registration number. | Methods section |

**Table S7.** PRISMA 2020 Checklist

| **Section and Topic** | **Item #** | **Checklist item** | **Location where item is reported** |
| --- | --- | --- | --- |
| **TITLE** | | |  |
| Title | 1 | Identify the report as a systematic review. | Provided |
| **ABSTRACT** | | |  |
| Abstract | 2 | See the PRISMA 2020 for Abstracts checklist. | Provided |
| **INTRODUCTION** | | |  |
| Rationale | 3 | Describe the rationale for the review in the context of existing knowledge. | Introduction paragraph |
| Objectives | 4 | Provide an explicit statement of the objective(s) or question(s) the review addresses. | Introduction paragraph |
| **METHODS** | | |  |
| Eligibility criteria | 5 | Specify the inclusion and exclusion criteria for the review and how studies were grouped for the syntheses. | Paragraph 2.2 |
| Information sources | 6 | Specify all databases, registers, websites, organisations, reference lists and other sources searched or consulted to identify studies. Specify the date when each source was last searched or consulted. | Paragraph 2.1 |
| Search strategy | 7 | Present the full search strategies for all databases, registers and websites, including any filters and limits used. | Paragraph 2.1 and Table S1 |
| Selection process | 8 | Specify the methods used to decide whether a study met the inclusion criteria of the review, including how many reviewers screened each record and each report retrieved, whether they worked independently, and if applicable, details of automation tools used in the process. | Paragraphs 2.2 and 2.3 |
| Data collection process | 9 | Specify the methods used to collect data from reports, including how many reviewers collected data from each report, whether they worked independently, any processes for obtaining or confirming data from study investigators, and if applicable, details of automation tools used in the process. | Paragraph 2.3 |
| Data items | 10a | List and define all outcomes for which data were sought. Specify whether all results that were compatible with each outcome domain in each study were sought (e.g. for all measures, time points, analyses), and if not, the methods used to decide which results to collect. | Paragraphs 2.2 and 2.3 |
|  | 10b | List and define all other variables for which data were sought (e.g. participant and intervention characteristics, funding sources). Describe any assumptions made about any missing or unclear information. | Not applicable as not foreseen |
| Study risk of bias assessment | 11 | Specify the methods used to assess risk of bias in the included studies, including details of the tool(s) used, how many reviewers assessed each study and whether they worked independently, and if applicable, details of automation tools used in the process. | Paragraph 2.4 |
| Effect measures | 12 | Specify for each outcome the effect measure(s) (e.g. risk ratio, mean difference) used in the synthesis or presentation of results. | Not applicable as not foreseen |
| Synthesis methods | 13a | Describe the processes used to decide which studies were eligible for each synthesis (e.g. tabulating the study intervention characteristics and comparing against the planned groups for each synthesis (item #5). | Paragraph 2.3 |
|  | 13b | Describe any methods required to prepare the data for presentation or synthesis, such as handling of missing summary statistics, or data conversions. | Paragraph 2.3 |
|  | 13c | Describe any methods used to tabulate or visually display results of individual studies and syntheses. | Paragraph 2.3 |
|  | 13d | Describe any methods used to synthesize results and provide a rationale for the choice(s). If meta-analysis was performed, describe the model(s), method(s) to identify the presence and extent of statistical heterogeneity, and software package(s) used. | Paragraph 2.3 |
|  | 13e | Describe any methods used to explore possible causes of heterogeneity among study results (e.g. subgroup analysis, meta-regression). | Not applicable as not foreseen |
|  | 13f | Describe any sensitivity analyses conducted to assess robustness of the synthesized results. | Not applicable as not foreseen |
| Reporting bias assessment | 14 | Describe any methods used to assess risk of bias due to missing results in a synthesis (arising from reporting biases). | Paragraph 2.4 |
| Certainty assessment | 15 | Describe any methods used to assess certainty (or confidence) in the body of evidence for an outcome. | Not applicable as not foreseen |
| **RESULTS** | | |  |
| Study selection | 16a | Describe the results of the search and selection process, from the number of records identified in the search to the number of studies included in the review, ideally using a flow diagram. | Paragraphs 3 and Figure 1 |
|  | 16b | Cite studies that might appear to meet the inclusion criteria, but which were excluded, and explain why they were excluded. | Figure 1 |
| Study characteristics | 17 | Cite each included study and present its characteristics. | Paragraph 3.1 |
| Risk of bias in studies | 18 | Present assessments of risk of bias for each included study. | Supplementary Material, Tables S2-S5 |
| Results of individual studies | 19 | For all outcomes, present, for each study: (a) summary statistics for each group (where appropriate) and (b) an effect estimate and its precision (e.g. confidence/credible interval), ideally using structured tables or plots. | Table 1 |
| Results of syntheses | 20a | For each synthesis, briefly summarise the characteristics and risk of bias among contributing studies. | Tables S2-S5 |
|  | 20b | Present results of all statistical syntheses conducted. If meta-analysis was done, present for each the summary estimate and its precision (e.g. confidence/credible interval) and measures of statistical heterogeneity. If comparing groups, describe the direction of the effect. | Table 1 |
|  | 20c | Present results of all investigations of possible causes of heterogeneity among study results. | Not applicable as not foreseen |
|  | 20d | Present results of all sensitivity analyses conducted to assess the robustness of the synthesized results. | Not applicable as not foreseen |
| Reporting biases | 21 | Present assessments of risk of bias due to missing results (arising from reporting biases) for each synthesis assessed. | Table 1, Paragraphs 3.1-3.3 |
| Certainty of evidence | 22 | Present assessments of certainty (or confidence) in the body of evidence for each outcome assessed. | Not applicable as not foreseen |
| **DISCUSSION** | | |  |
| Discussion | 23a | Provide a general interpretation of the results in the context of other evidence. | Paragraph 4 |
|  | 23b | Discuss any limitations of the evidence included in the review. | Paragraph 4 |
|  | 23c | Discuss any limitations of the review processes used. | Paragraph 4 |
|  | 23d | Discuss implications of the results for practice, policy, and future research. | Paragraph 4 |
| **OTHER INFORMATION** | | |  |
| Registration and protocol | 24a | Provide registration information for the review, including register name and registration number, or state that the review was not registered. | Abstract and methods |
|  | 24b | Indicate where the review protocol can be accessed, or state that a protocol was not prepared. | Abstract and methods |
|  | 24c | Describe and explain any amendments to information provided at registration or in the protocol. | Not applicable |
| Support | 25 | Describe sources of financial or non-financial support for the review, and the role of the funders or sponsors in the review. | Not applicable as not foreseen |
| Competing interests | 26 | Declare any competing interests of review authors. | Conflict of interest statement |
| Availability of data, code and other materials | 27 | Report which of the following are publicly available and where they can be found: template data collection forms; data extracted from included studies; data used for all analyses; analytic code; any other materials used in the review. | Supplementary materials, Paragraph 2. Methods |
